# Pre-Deformation-Assisted Retrogression and Re-Aging Treatment for Achieving Strength–Corrosion Synergy in 2195 Al–Cu–Li Alloy: Insights into Cu Redistribution and Grain Boundary Evolution

**DOI:** 10.3390/ma19163464

**Published:** 2026-08-16

**Authors:** Boyuan Zhou, Sheng Li, Shaoxuan Wu, Yao Li, Zhe Liu, Hongqun Tang, Wenbo Liu

**Affiliations:** 1 School of Resources, Environment and Materials, Guangxi University, Nanning 530004, China; 13607628630@163.com (B.Z.); 19338602056@163.com (S.L.); 17677607756@163.com (S.W.); ly18076791294@163.com (Y.L.); tanghongqun@126.com (H.T.); 2 School of Materials Science and Engineering, Central South University, Changsha 410083, China; 13159617283@163.com; 3 Guangxi Key Laboratory of Processing for Non-Ferrous Metals and Featured Materials, Guangxi University, Nanning 530004, China

**Keywords:** Al–Cu–Li alloys, RRA treatment, Cu redistribution, strength–corrosion synergy

## Abstract

Al–Cu–Li alloys face an intrinsic trade-off between precipitation strengthening and intergranular corrosion susceptibility. This trade-off restricts the simultaneous improvement of their strength and corrosion resistance. In this work, a pre-deformation-assisted retrogression and re-aging (RRA) strategy was developed to regulate Cu redistribution and balance the mechanical and corrosion properties of 2195 Al–Cu–Li alloy. The results show that short-time retrogression maintains effective strengthening while limiting Cu depletion near grain boundaries, thereby achieving an improved strength–corrosion balance under the investigated processing conditions. The RRA-5 min sample exhibited the optimum performance, with a yield strength of 533 MPa, ultimate tensile strength of 562.6 MPa, and polarization resistance of 19,605 Ω·cm^2^. Prolonged retrogression promoted Cu redistribution toward grain boundaries, potentially promoting precipitate-free zone (PFZ) evolution, electrochemical heterogeneity, and intergranular corrosion susceptibility. XPS analysis revealed that the corrosion film consisted mainly of Al oxides and hydroxides, Li- and Mg-containing compounds, and Cu-enriched species, suggesting selective dissolution and corrosion film reconstruction during exposure. The coupled Cu diffusion–grain boundary absorption model predicted the evolution tendency of PFZ widening with increasing retrogression time, which was consistent with the experimental trend. This study provides insights into the regulation of Cu redistribution through RRA processing for achieving strength–corrosion synergy in aerospace Al–Cu–Li alloys under the present processing conditions.

## 1. Introduction

The rapid development of aerospace and deep-space exploration technologies has imposed increasingly stringent requirements on advanced structural materials, which are expected to simultaneously possess high specific strength, excellent damage tolerance, and superior corrosion resistance [1,2,3]. Among advanced aerospace aluminum alloys, 2195 Al–Cu–Li alloy (Al–Cu–Li–Mg–Ag) has attracted extensive attention due to its low density and outstanding specific modulus, and has become one of the most promising candidates for next-generation aerospace structures [4,5,6,7]. However, a long-standing challenge associated with the engineering application of this alloy is the intrinsic trade-off between mechanical strength and corrosion resistance [8,9,10]. Conventional aging treatments, such as T6 and T8 tempers, can generate a high density of T_1_ (Al_2_CuLi) strengthening precipitates and provide excellent strengthening effects, but they usually exhibit high susceptibility to intergranular corrosion due to unfavorable grain boundary precipitation characteristics [11,12]. In contrast, over-aging treatments (e.g., T7 temper) can improve corrosion resistance by modifying grain boundary precipitate morphology and reducing electrochemical heterogeneity, but inevitably result in significant strength degradation [13,14].

Previous studies have demonstrated that retrogression and re-aging (RRA) treatment is an effective approach for overcoming the strength–corrosion resistance trade-off in precipitation-strengthened aluminum alloys. Zhang et al. reported that RRA-treated 7A99 aluminum alloy exhibited the largest grain boundary precipitate size and spacing, resulting in the best stress corrosion cracking (SCC) resistance, while maintaining relatively high strength and hardness [15]. Zhang et al. further investigated the effects of different retrogression cooling strategies during RRA treatment on the microstructural evolution, mechanical properties, and intergranular corrosion resistance of T′/η′-strengthened Al–Zn–Mg–Cu alloys [16]. Guo et al. found that compared with the conventional T6 temper, RRA treatment of 2024 aluminum alloy significantly reduced the volume fraction of coarse second phases while increasing the fraction of strengthening precipitates [17]. Furthermore, Guo et al. demonstrated that for Al–Cu–Mg alloys, when pre-aging was conducted in the under-aged to peak-aged stage, subsequent re-aging performed after retrogression at the minimum hardness stage could achieve the highest hardness after RRA treatment [18]. These studies indicate that the evolution of precipitate morphology and distribution during RRA treatment plays a critical role in controlling the mechanical and corrosion properties of aluminum alloys.

For Al–Cu–Li alloys, precipitation behavior is governed by complex competitive interactions among multiple phases, and the Cu/Li atomic ratio and aging parameters significantly influence precipitation evolution. Therefore, extensive efforts have been devoted to understanding the effects of pre-deformation treatments on the microstructure, mechanical properties, and corrosion resistance of Al–Cu–Li alloys. Zou et al. compared the effects of 5% pre-stretching and 18% pre-rolling deformation on the aging precipitation behavior, precipitate characteristics, and high-temperature mechanical properties of 2195 Al–Cu–Li alloy, demonstrating that pre-deformation accelerated aging kinetics and improved the overall mechanical performance [19]. Yue et al. systematically investigated the effects of rolling reduction and rolling temperature on the mechanical properties and corrosion behavior of AA2195 aluminum alloy plates, and found that the corrosion resistance of room-temperature rolled samples decreased with increasing rolling reduction [20]. Xu et al. revealed that the intergranular corrosion and exfoliation corrosion susceptibility of 2195 Al–Li alloy mainly originated from micro-galvanic coupling among T_1_ phase, θ′ phase, and grain boundary precipitate-free zones (PFZs). In addition, surfaces parallel to the rolling direction exhibited higher susceptibility to exfoliation corrosion than those perpendicular to the rolling direction [21]. Yao et al. investigated the effects of different low-temperature deformation combined with single-stage aging treatments on the microstructure and mechanical properties of 2195 Al–Li alloy and established optimized processing parameters for microstructure regulation [22]. Although considerable progress has been achieved regarding the influence of RRA parameters on microstructural evolution, mechanical properties, and corrosion resistance of Al–Cu–Li alloys, as well as the role of pre-treatment processes in regulating single- or multi-stage aging behavior, the synergistic effects of pre-deformation combined with RRA treatment on the simultaneous enhancement of mechanical properties and corrosion resistance remain insufficiently understood. Furthermore, the influence of PFZ morphology and width on mechanical degradation and corrosion damage evolution, as well as the underlying corrosion mechanisms, have not yet been fully clarified [23,24,25,26].

Based on the above considerations, this study combines experimental investigations, COMSOL Multiphysics simulations, and mechanistic analysis to systematically investigate the microstructural evolution and property variations of 2195 Al–Cu–Li alloy subjected to solution treatment, 5% room-temperature rolling, and subsequent RRA treatment. Tensile testing, Vickers hardness measurements, electrochemical corrosion tests, and intergranular corrosion (IGC) evaluations were performed to comprehensively assess the mechanical properties and corrosion resistance. X-ray photoelectron spectroscopy (XPS) depth profiling at different sputtering depths (0–30 nm) was conducted to characterize surface Cu redistribution and investigate the relationship between PFZ-associated solute depletion and surface chemical evolution. Meanwhile, fracture surface SEM observations and microscopic analysis of corrosion morphology and penetration depth were employed to elucidate the influence of PFZ evolution on crack initiation and propagation behavior. This work aims to achieve simultaneous improvement of strength and corrosion resistance in 2195 Al–Cu–Li alloy through the synergistic combination of solution treatment, 5% room-temperature rolling, and RRA processing, and to establish the intrinsic correlation between PFZ evolution, mechanical properties, and corrosion performance, thereby providing a multiscale understanding of the relationship between microstructural characteristics and macroscopic properties.

## 2. Materials and Methods

**Figure 1:**
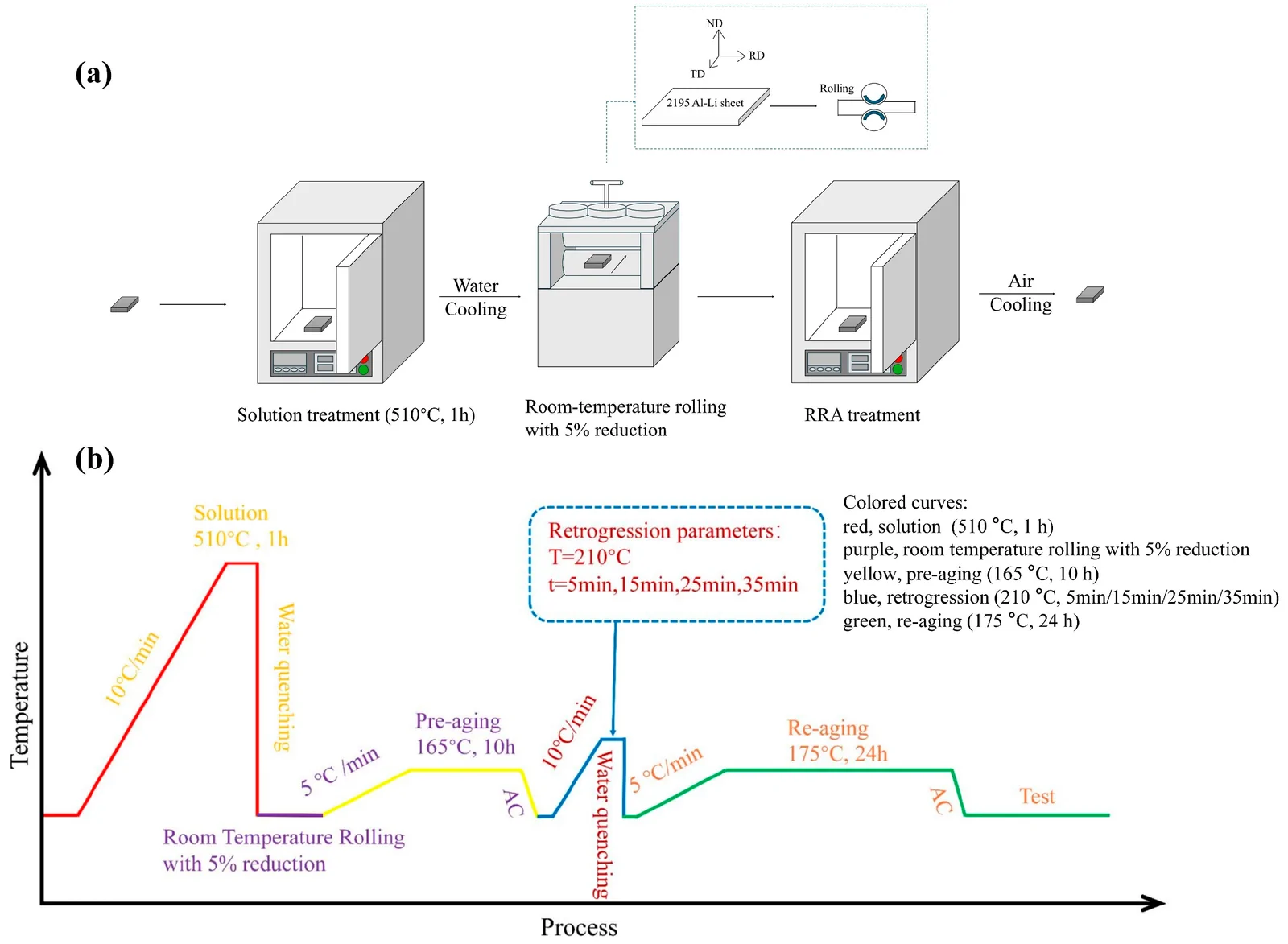
Experimental flowchart (**a**), experimental process routes for 2195 Al–Cu–Li alloy (**b**).

The 2195 Al–Li alloy plates used in this study were selected as the research material, and their chemical composition is listed in Table 1. The O-state 2195 Al–Cu–Li alloy was used as the starting material in this study. Because the primary objective was to evaluate the effect of retrogression time within a fixed pre-deformation-assisted RRA processing route, the microhardness, tensile, and electrochemical properties were primarily compared among samples subjected to the same thermomechanical processing history, with retrogression time as the only variable. The plates were solution-treated at 510 °C for 1 h, followed by water quenching. Subsequently, room-temperature rolling was performed using a laboratory rolling mill with a thickness reduction of 5%. After deformation, the rolled plates were subjected to retrogression and re-aging (RRA) treatment, in which the retrogression times were set to 5, 15, 25, and 35 min [27]. All other processing parameters were kept constant, including solution treatment at 510 °C for 1 h, 5% room-temperature pre-deformation, pre-aging at 165 °C for 10 h, and re-aging at 175 °C for 24 h. The schematic illustration of the experimental procedure and the corresponding heat treatment schedule for the 2195 Al–Cu–Li alloy are presented in Figure 1a,b respectively.

Vickers hardness measurements were performed using a NVM-1000A nanoindentation hardness tester. To ensure experimental reproducibility, three independent measurements were conducted for each processing condition, and the average value was taken as the final hardness result.

Electrochemical impedance spectroscopy (EIS) and potentiodynamic polarization measurements were employed to evaluate the electrochemical corrosion behavior of the alloys. Prior to electrochemical testing, the specimens were immersed in 3.5 wt.% NaCl solution for 1 h to stabilize the open-circuit potential (OCP). Electrochemical measurements were carried out using a CHI750E electrochemical workstation in a conventional three-electrode configuration, with a saturated calomel electrode (SCE) as the reference electrode, a platinum plate as the counter electrode, and the specimen as the working electrode. The exposed area of the working electrode was 1 cm^2^, and 3.5 wt% NaCl solution was used as the electrolyte. The potentiodynamic polarization curves were recorded within a potential range of −1.2 to −0.4 V (vs. SCE) at a scan rate of 1 mV/s. EIS measurements were conducted over a frequency range from 100 kHz to 0.01 Hz. Three parallel measurements were performed for each processing condition to ensure the reliability of the electrochemical results.

**Figure 2:**
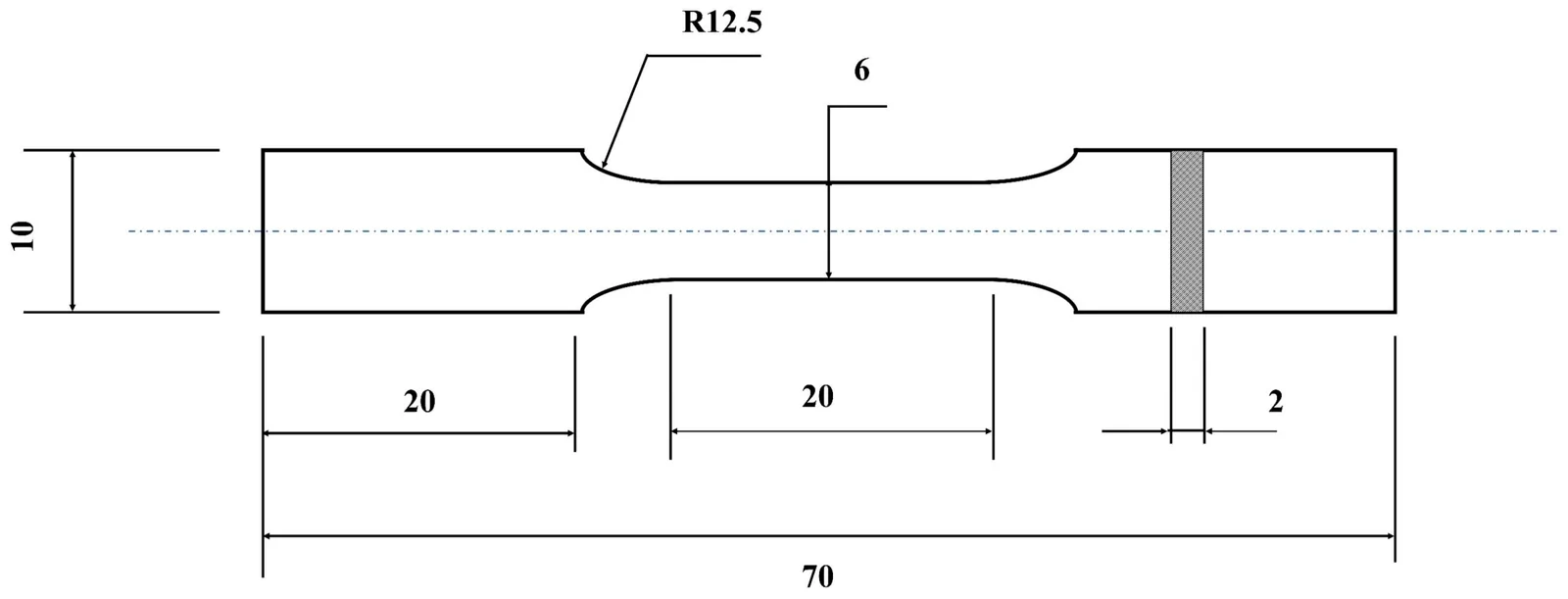
Room temperature tensile sample.

Room-temperature uniaxial tensile tests were conducted using a DWD-2000 computer-controlled universal testing machine. Three replicate specimens were tested for each processing condition, and the specimen geometry and dimensions are shown in Figure 2. All specimens were machined from the rolled plate via wire electrical discharge machining and subsequently polished to obtain uniform and smooth surface finishes. The tensile loading direction was parallel to the rolling direction (RD) of the plate to maintain consistency with the pre-deformation orientation. After tensile testing, the fracture morphologies were examined using scanning electron microscopy (SEM) to characterize the fracture features. The SEM observations were performed at an accelerating voltage of 15 kV and a working distance of 60 μm.

IGC tests were performed according to the Chinese national standard GB/T 7998–2023 [28]. The specimens were machined into dimensions of 40 mm × 25 mm × 2 mm, and three parallel samples were tested for each processing condition. The IGC solution was prepared by dissolving 57 g NaCl and 10 mL H_2_O_2_ in 1 L deionized water. After corrosion exposure, the specimens were rinsed with deionized water and dried. The corrosion morphology on the cross-sections of the specimens was subsequently examined using an optical microscope.

X-ray photoelectron spectroscopy (XPS) (Thermo Scientific K-Alpha, Thermo Fisher Scientific, Waltham, MA, USA) was conducted using monochromatic Al Kα radiation (hν = 1486.6 eV) to characterize the corrosion products on the RRA-5 min sample at different sputtering depths (0, 10, 20, and 30 nm). To minimize the influence of local compositional variations, three randomly selected areas were analyzed at each depth, and the averaged results were used for elemental composition analysis. The spectra were calibrated using the C 1s peak at 284.8 eV, processed by Shirley background subtraction, and fitted using Avantage 2026 software based on standard binding energy assignments.

## 3. Results and Discussion

### 3.1. Mechanical Properties

**Figure 3:**
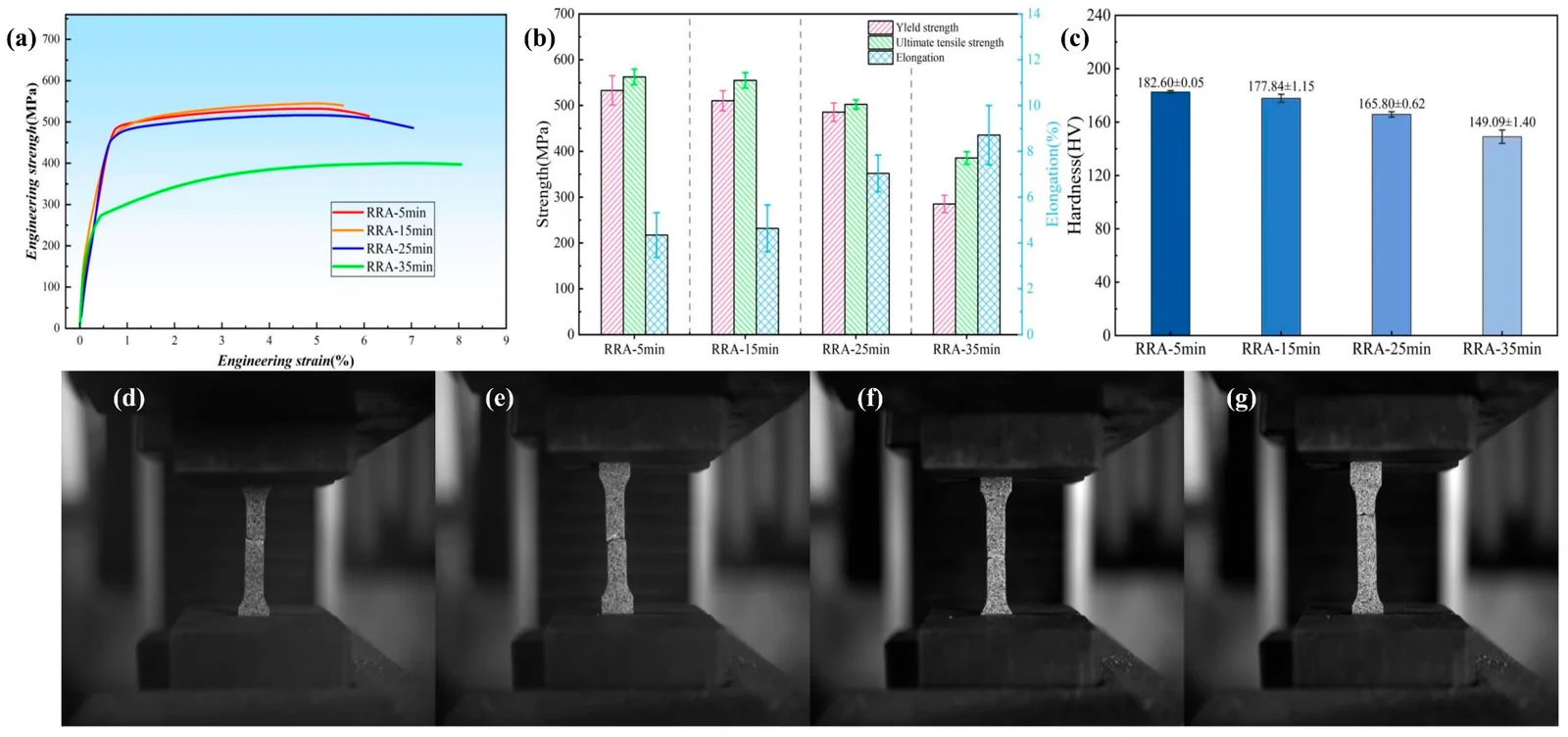
The engineering stress–strain curves of 2195 Al–Cu–Li alloy (**a**) mechanical properties of 2195 Al–Cu–Li alloys (**b**), hardness value (**c**), side-view images of the 2195 Al–Cu–Li alloys tensile specimens after fracture, 210 °C retrogression for 5 min (**d**), 15 min (**e**), 25 min (**f**), 35 min (**g**).

Figure 3a presents the engineering stress–strain curves of the 2195 Al–Cu–Li alloy under different RRA treatment conditions. All samples exhibit a similar linear elastic response at the initial deformation stage, followed by a pronounced work-hardening stage. The RRA-5 min sample shows the highest flow stress level, with the maximum yield strength and ultimate tensile strength, although its elongation is relatively limited. With increasing RRA time to 15 and 25 min, the overall stress–strain curves gradually shift downward, corresponding to a continuous decrease in yield strength and ultimate tensile strength, while the capacity for uniform plastic deformation is progressively enhanced. When the RRA duration is further extended to 35 min, the alloy exhibits the lowest strength but significantly improved elongation, indicating a transition from a strength-dominated state toward a ductility-dominated state.

The statistical mechanical properties shown in Figure 3b further confirm this evolution trend. Both yield strength and ultimate tensile strength exhibit a monotonic decrease with increasing RRA time, whereas elongation gradually increases. This behavior suggests that prolonged retrogression and re-aging treatment continuously weakens precipitation strengthening and reduces the resistance to dislocation motion, resulting in decreased load-bearing capability and enhanced plastic deformation capacity.

Figure 3c illustrates the variation in microhardness of the 2195 Al–Cu–Li alloy subjected to different RRA treatment times. Overall, the hardness exhibits a continuous decreasing trend with increasing RRA duration. Specifically, the RRA-5 min sample presents the highest hardness value of 182.60 ± 0.05 HV. After increasing the treatment time to 15 min, the hardness slightly decreases to 177.84 ± 1.15 HV, while maintaining a relatively high level. Further extension of the RRA time to 25 min results in a more pronounced decrease in hardness to 165.80 ± 0.62 HV. When the RRA duration reaches 35 min, the hardness decreases to the minimum value of 149.09 ± 1.40 HV. This result indicates that prolonged RRA treatment promotes precipitate coarsening and solute depletion near grain boundaries, thereby weakening precipitation strengthening and leading to a continuous reduction in hardness.

This evolution trend is consistent with previous studies on the RRA treatment of aluminum alloys. Zhang et al. investigated the RRA behavior of 7050 aluminum alloy and reported that, with prolonged retrogression time, the strengthening elements in the supersaturated solid solution undergo progressive redistribution, accompanied by partial dissolution, coarsening, and stabilization of the original strengthening structures. These microstructural changes gradually reduce the contribution of precipitation strengthening, resulting in decreased hardness and an eventual transition toward an over-aged state [29]. Similarly, in the present study, the continuous decrease in hardness of the 2195 Al–Cu–Li alloy with increasing RRA time reflects the typical softening behavior during the retrogression stage. This phenomenon is mainly attributed to enhanced solute diffusion during thermal treatment, reduced stability of strengthening structures, and the gradual weakening of the resistance to plastic deformation, leading to a decrease in the material’s ability to resist localized deformation. Notably, compared with conventional RRA-treated 2195 Al–Cu–Li alloys, the present alloy achieves a relatively high strength level within a shorter retrogression duration after 5% room-temperature rolling. This behavior can be attributed to the high density of dislocations introduced by pre-deformation, which provides fast diffusion pathways and heterogeneous nucleation sites during subsequent aging, thereby accelerating solute redistribution and the formation of strengthening structures [30]. Therefore, pre-deformation-assisted RRA treatment not only modifies the initial defect structure of the alloy but also regulates the subsequent microstructural evolution kinetics during thermal treatment [31].

Figure 3d–g further illustrates the evolution of deformation localization behavior under different RRA conditions. The RRA-5 min and RRA-15 min samples exhibit limited necking regions, indicating localized plastic deformation and stronger deformation constraints before fracture. This behavior is associated with their higher strength levels, where enhanced microstructural strengthening restricts dislocation motion and uniform plastic flow, facilitating strain concentration and crack propagation. With prolonged RRA time, the necking region gradually expands, indicating improved deformation compatibility and a greater capacity for uniform plastic deformation. The reduced deformation resistance under longer retrogression conditions promotes dislocation slip and plastic accommodation, consistent with the increased elongation observed in tensile tests. These results demonstrate that RRA treatment effectively tailors the deformation behavior of the 2195 Al–Cu–Li alloy from a high-strength state toward a more ductile deformation state [32].

**Figure 4:**
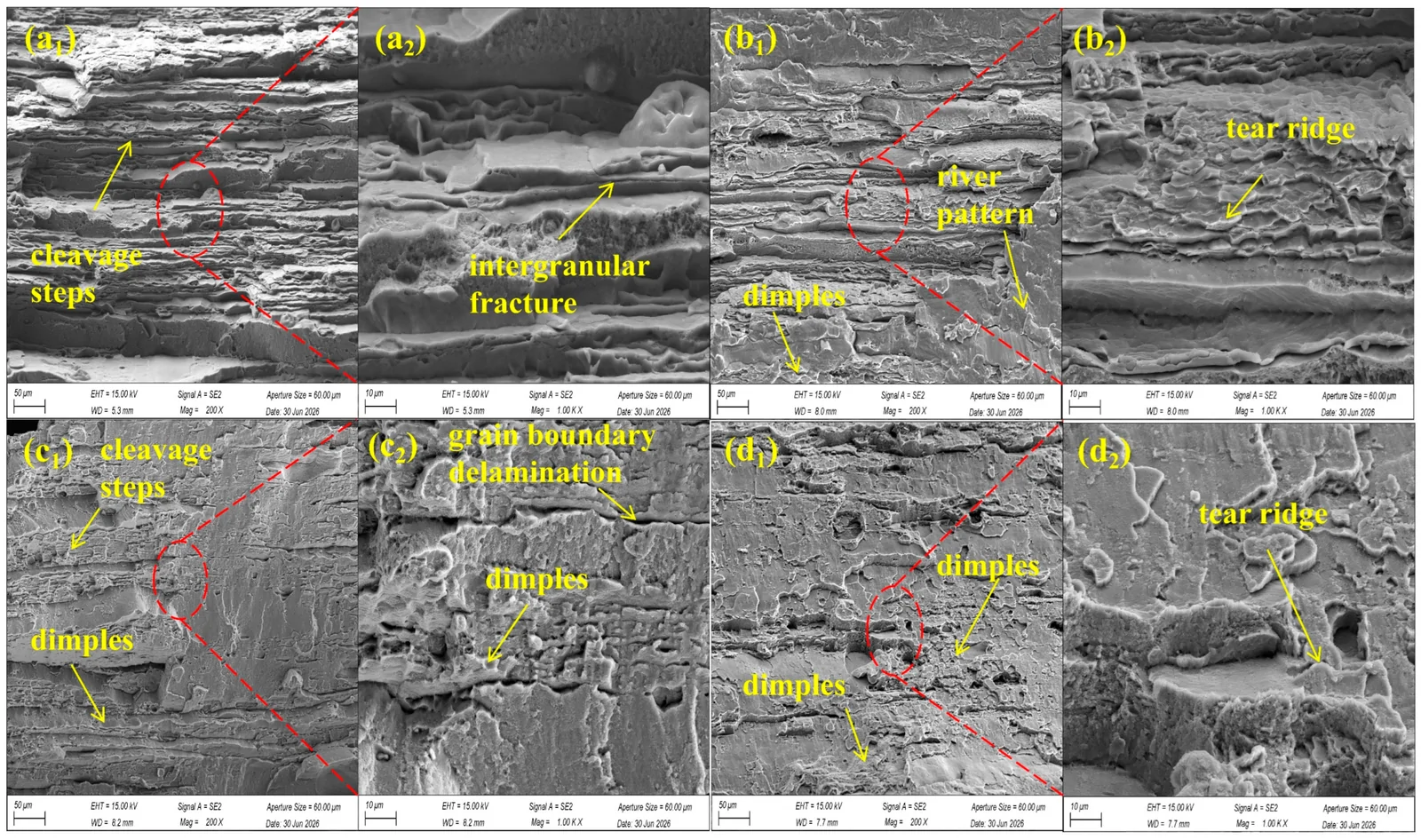
SEM micrographs of tensile fracture surface of 2195 Al–Cu–Li alloy under different retrogression conditions: (**a_1_**,**a_2_**) 5 min, (**b_1_**,**b_2_**) 15 min, (**c_1_**,**c_2_**) 25 min, (**d_1_**,**d_2_**) 35 min.

To elucidate the tensile fracture mechanisms of the 2195 Al–Cu–Li alloy subjected to different RRA treatment times, scanning electron microscopy (SEM) observations of the fracture surfaces were performed, as shown in Figure 4. The fracture characteristics were further correlated with the corresponding mechanical properties. Under the RRA-5 min condition (a_1_,a_2_), the fracture surface exhibits a limited number of dimples accompanied by obvious cleavage steps and quasi-cleavage features, indicating a mixed fracture mode dominated by transgranular brittle fracture characteristics. This behavior corresponds to the highest ultimate tensile strength (562.6 MPa) and yield strength (533 MPa) obtained under this condition. The fine and densely distributed strengthening features effectively enhance matrix strengthening; however, they simultaneously restrict plastic deformation accommodation, facilitating rapid crack propagation under tensile loading. When the RRA time is extended to 15 min (b_1_,b_2_), the fracture surface contains an increased number of dimples, accompanied by tearing ridges and localized river patterns. These features suggest a gradual transition from quasi-cleavage-dominated fracture toward a more ductile fracture mode. This transition is consistent with the tensile behavior, where relatively high strength is maintained while elongation is slightly improved. For the RRA-25 min sample (c_1_,c_2_), the fracture surface is mainly characterized by uniformly distributed dimples, together with localized grain boundary separation and delamination features. This indicates that microvoid nucleation and growth gradually become the dominant fracture mechanism. Although the ultimate tensile strength decreases to 502.4 MPa, the elongation increases significantly to 7.04%, demonstrating an improved balance between strength and ductility. With further extension of the RRA time to 35 min (d_1_,d_2_), the fracture surface exhibits numerous large and deep dimples accompanied by pronounced tearing ridges, while quasi-cleavage characteristics almost disappear. The fracture process is primarily controlled by ductile microvoid coalescence. Correspondingly, this condition exhibits the lowest ultimate tensile strength (385.5 MPa) but the highest elongation (8.71%), which agrees well with the mechanical property results, shows reduced strength and enhanced ductility.

Increasing the RRA treatment time gradually transforms the fracture mode of the 2195 Al–Cu–Li alloy from a relatively brittle fracture dominated by quasi-cleavage characteristics to a ductile fracture governed by microvoid coalescence. This transition is associated with the evolution of strengthening features, reduced grain boundary strengthening effects, and increased softening of PFZ-related regions, ultimately leading to a transformation from a high-strength and low-ductility state toward a low-strength and high-ductility state.

### 3.2. Corrosion Behavior

**Figure 5:**
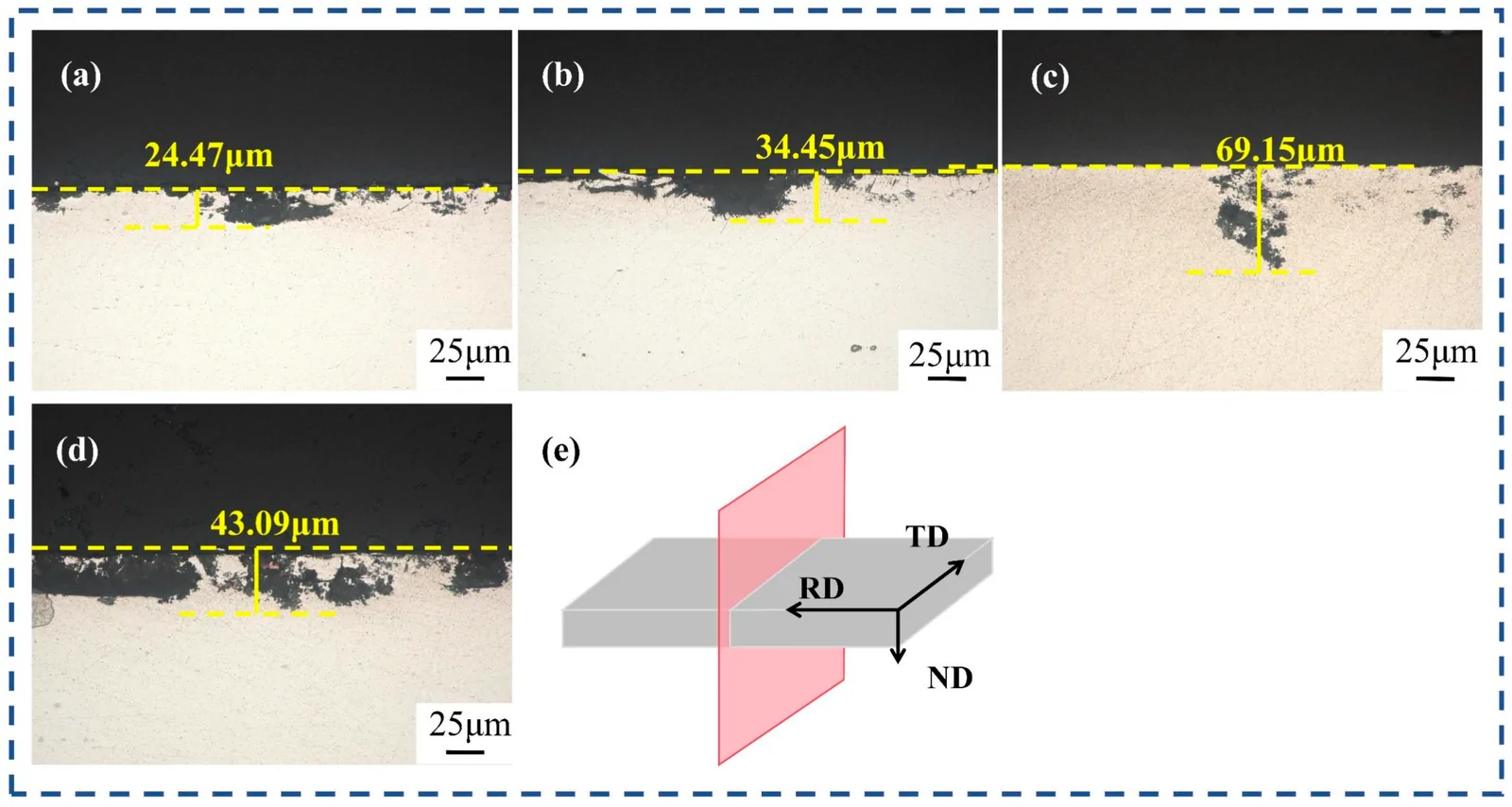
Schematics of corrosion testing samples (**e**) and typical cross-sectional (TD-ND) corrosion morphologies of samples (**a**) RRA-5 min, (**b**) RRA-15 min, (**c**) RRA-25 min, (**d**) RRA-35 min after being exposed for 6 h to a solution of 57 g/L NaCl and 10 mL/L H_2_O_2_ at 30 ± 3 °C.

**Figure 6:**
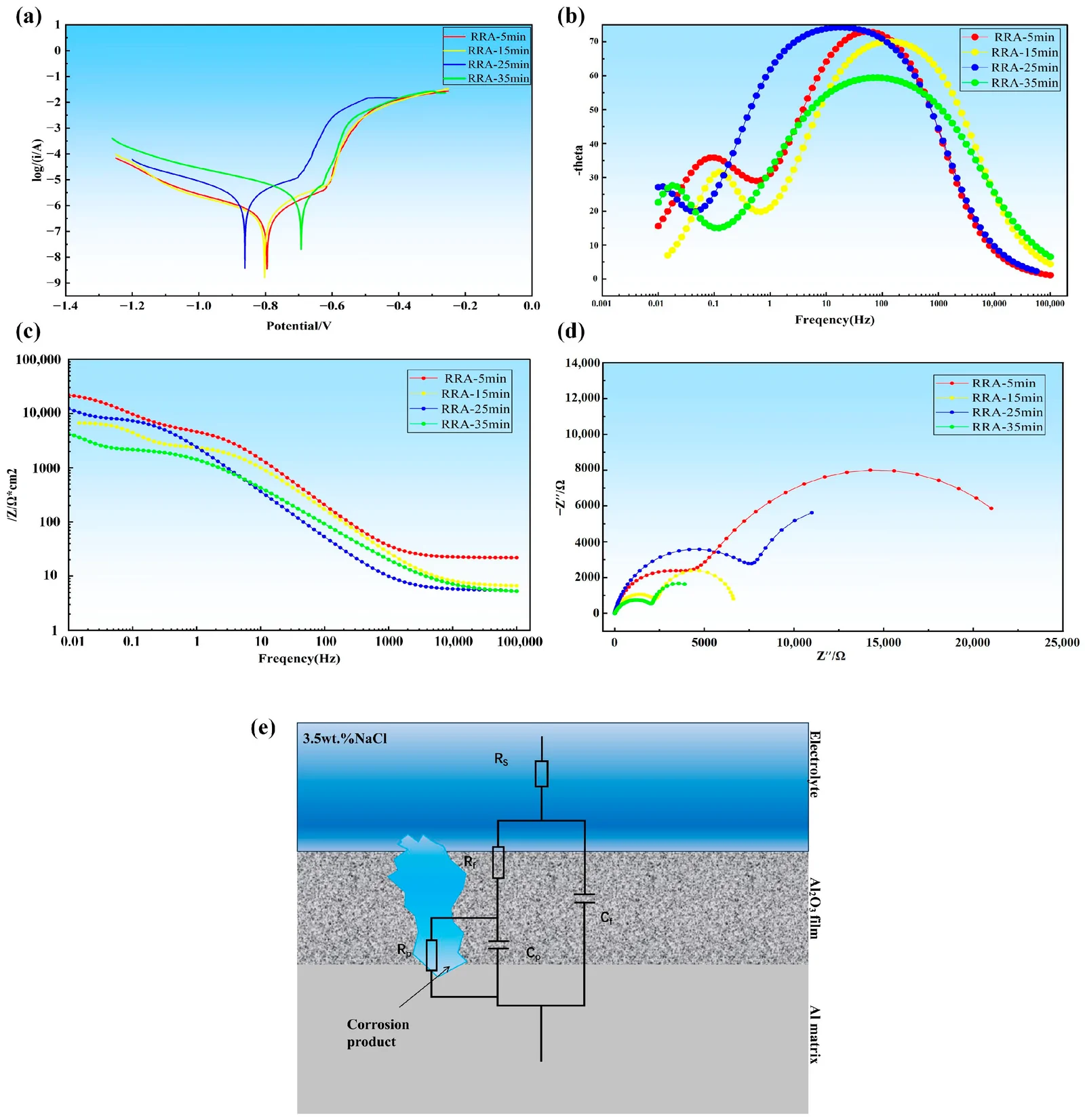
The electrochemical impedance spectroscopy of 2195 Al–Cu–Li alloy with different retrogression conditions in NaCl solution:Tafel diagram (**a**), Bode diagram (**b**,**c**), Nyquist diagram (**d**), equivalent circuit (**e**).

Figure 5 shows the cross-sectional morphologies after intergranular corrosion (IGC) testing, while Figure 6 presents the electrochemical corrosion behavior of the 2195 Al–Cu–Li alloy after different RRA treatments (5–35 min), including polarization curves, EIS spectra, equivalent circuit fitting, and electrochemical parameters. The combined results indicate that RRA treatment significantly affects both general electrochemical corrosion behavior and localized corrosion susceptibility, which are closely related to the evolution of precipitate characteristics, grain boundary structures, and solute redistribution.

As shown in Figure 5, the IGC penetration depth varies non-monotonically with increasing RRA duration. The RRA-5 min sample exhibits the lowest corrosion depth (24.47 μm), indicating relatively low susceptibility to grain boundary corrosion. With increasing RRA time to 15 min, the corrosion depth slightly increases to 34.45 μm. A further increase to 25 min results in the maximum corrosion penetration depth of 69.15 μm, suggesting enhanced localized corrosion along susceptible grain boundary regions. Interestingly, the corrosion depth decreases to 43.09 μm for the RRA-35 min sample, indicating that the influence of RRA duration on IGC behavior is not simply linear and involves the combined effects of precipitate evolution and grain boundary characteristics.

The polarization results in Figure 6a further reveal the variation in corrosion kinetics. As summarized in Table 2, the RRA-5 min sample exhibits the most positive Ecorr (−0.7933 V) and the lowest Icorr (3.89 × 10^−7^ A·cm^−2^), demonstrating the highest electrochemical stability. After extending the RRA time to 15 min, Ecorr slightly decreases to −0.8120 V, while Icorr remains almost unchanged (4.14 × 10^−7^ A·cm^−2^), indicating limited variation in corrosion kinetics. When the RRA time increases to 25 min, Ecorr shifts negatively to −0.8660 V and Icorr increases to 1.90 × 10^−6^ A·cm^−2^, suggesting accelerated electrochemical reactions. Although the RRA-35 min sample exhibits a more positive Ecorr (−0.6494 V), its relatively high Icorr (1.49 × 10^−6^ A·cm^−2^) indicates that corrosion potential alone cannot fully reflect the localized corrosion resistance.

The EIS results in Figure 6b–d show a similar evolution trend. As listed in Table 3, The RRA-5 min sample exhibits the highest low-frequency impedance modulus and the largest capacitive semicircle diameter, corresponding to the highest polarization resistance (Rp = 19,605 Ω·cm^2^). This indicates improved interfacial resistance and enhanced protection of the surface film. With increasing RRA duration, the impedance response gradually decreases, accompanied by a reduction in phase angle maximum, suggesting increased interfacial electrochemical activity. The equivalent circuit fitting results further confirm that the decreased Rp values for RRA-25 min and RRA-35 min samples are associated with reduced resistance against charge transfer.

The variation in corrosion behavior can be related to the microstructural evolution during RRA treatment. During short-term retrogression, the relatively stable precipitate and grain boundary structures contribute to lower electrochemical heterogeneity, resulting in improved corrosion resistance. With prolonged RRA treatment, solute redistribution and changes in grain boundary precipitate characteristics may increase the difference between grain boundary regions and the Al matrix, thereby promoting localized corrosion susceptibility. This interpretation is consistent with the increased Icorr values and the maximum IGC penetration depth observed for the RRA-25 min condition.

However, the reduced IGC depth of the RRA-35 min sample compared with the RRA-25 min sample suggests that corrosion propagation is not solely governed by grain boundary solute redistribution. With prolonged retrogression, further precipitation evolution, possibly approaching an over-aged state, may alter local electrochemical heterogeneity and affect grain boundary corrosion behavior [29]. Combined with electrochemical measurements, XPS analysis, and cross-sectional observations, this result indicates that corrosion resistance is controlled by the coupled effects of surface film evolution, solute redistribution, and precipitation-related changes, which require further characterization for confirmation.

In summary, the combined electrochemical measurements and IGC observations demonstrate that RRA duration plays an important role in regulating the corrosion behavior of the 2195 Al–Cu–Li alloy. The RRA-5 min condition provides the best corrosion resistance among the investigated conditions and the corrosion resistance exhibits a general decreasing tendency with increasing RRA time, although localized corrosion susceptibility shows a non-monotonic variation. These results highlight the importance of controlling the retrogression time to achieve a balance between strength and corrosion resistance in pre-deformation-assisted RRA-treated Al–Cu–Li alloys.

### 3.3. Conceptual Modeling of Cu Redistribution

A reduced-order diffusion–sink model was established using COMSOL Multiphysics 6.3 to describe the evolution of the PFZs in 2195 Al–Cu–Li alloy during retrogression and re-aging (RRA) treatment. The model couples solute diffusion with grain boundary absorption behavior to capture the redistribution characteristics of Cu atoms under complex thermo-mechanical processing conditions.

To reduce computational complexity and obtain converged solutions, several assumptions were adopted:

(1) The three-dimensional grain structure was simplified as a two-dimensional circular grain with a radius of 10 μm, assuming radial symmetry of PFZ formation along the grain boundary.
(2) The transient heat transfer processes during heating and cooling between different aging stages were neglected, and the temperature of each stage was assumed to instantaneously reach the preset value. The effects of heating and cooling during retrogression were incorporated through an empirical smoothing function applied to the diffusion coefficient.
(3) After solution treatment, the Cu concentration inside the grain was assumed to be uniformly distributed.
(4) The Cu concentration was considered sufficiently low compared with the Al matrix, allowing the dilute solution approximation and application of Fick’s second law.
(5) The grain boundary was simplified as a solute sink with finite absorption capacity, where the absorption rate was proportional to the solute concentration at the boundary (N = kc), while the complex atomic-scale grain boundary structures were neglected.
(6) The diffusion coefficient was assumed to be isotropic, and the influence of crystallographic texture on diffusion anisotropy was ignored.
(7) Lattice diffusion, grain boundary diffusion, and dislocation-assisted diffusion were integrated into a single effective diffusion coefficient without explicit separation of individual diffusion contributions.

It should be noted that, owing to the absence of direct nanoscale microstructural characterization, the present diffusion–sink model is intended to provide a qualitative description of the evolution tendency of Cu redistribution and PFZ development during RRA treatment, rather than a quantitative prediction of the exact microstructural features. Accordingly, the simulation results should be interpreted as trend analysis that complements the experimental observations.

Although Cu diffusion in the Al matrix generally follows an Arrhenius relationship, the actual diffusion behavior during RRA treatment can deviate from ideal lattice diffusion due to the presence of high dislocation density, subgrain structures, and non-equilibrium grain boundaries, which provide short-circuit diffusion pathways. Therefore, an effective diffusion coefficient was introduced to comprehensively describe the combined contributions of lattice diffusion and short-circuit diffusion mechanisms, with stage-dependent corrections applied according to different thermal treatment conditions. During pre-aging, solute clustering and the formation of GP zones/fine precipitates reduce the amount of mobile Cu atoms and enhance solute trapping, thereby suppressing diffusion. During retrogression, defect structure rearrangement promotes solute migration pathways and increases diffusion capability. During re-aging, precipitate growth enhances Cu trapping and reduces the effective diffusion coefficient [33,34]. Accordingly, the effective diffusion coefficients during pre-aging, retrogression, and re-aging were expressed as:

(1) $$D_{\mathrm{pre}}^{\mathrm{eff}}=D_{\mathrm{Arr}}\;(T)\cdot f_{\mathrm{micro}}$$

where $f_{\mathrm{micro}}<1$.

(2) $$D_{\mathrm{reg}}^{\mathrm{eff}}{=D}_{\mathrm{Arr}}\;(T)\cdot f_{\mathrm{micro}}$$

where $f_{\mathrm{micro}}>1$.

(3) $$D_{\mathrm{re}}^{\mathrm{eff}}{=D}_{\mathrm{Arr}}\;(T)\cdot f_{\mathrm{micro}}$$

where $f_{\mathrm{micro}}<1$.

The diffusion behavior of Cu atoms in the Al matrix was described using Fick’s second law:

(4) $$\frac{\partial c}{\partial t}=\nabla \cdot \left(D_{\mathrm{eff}}\nabla c\right)$$

The diffusion coefficient adopted in this model does not strictly represent the ideal Arrhenius-type lattice diffusion coefficient. Instead, it is defined as an effective diffusion coefficient that incorporates the combined effects of lattice diffusion, grain boundary diffusion, and deformation-induced dislocation-assisted diffusion during room-temperature rolling. These diffusion mechanisms were not explicitly separated but integrated into a unified diffusion term. The effective diffusion parameters were calibrated within physically reasonable ranges reported in the literature to reproduce PFZ evolution behavior, which has been widely adopted in microstructure-scale PFZ simulations and precipitation-related modeling studies [35,36]. To account for the different thermomechanical conditions during RRA treatment, the effective diffusion coefficient was defined as a stage-dependent parameter:

(5) $$D_{\mathrm{eff}}=\left(D_{\mathrm{pre}}^{\mathrm{eff}}I_{1}+D_{\mathrm{reg}}^{\mathrm{eff}}I_{2}+D_{\mathrm{re}}^{\mathrm{eff}}I_{3}\right)\left(1+\alpha \varepsilon \right)$$

where $I_{1},\;I_{2}\;and\;I_{3}$ are indicator functions corresponding to the pre-aging, retrogression, and re-aging stages, respectively. The term 1+αε represents the enhancement of atomic mobility induced by cold rolling deformation.

In this model, the grain boundary was treated as a fast diffusion pathway with finite solute absorption capability, and the parameter k was introduced to characterize the kinetic strength of grain boundary absorption [37,38]. The grain boundary flux was described using first-order absorption kinetics:

(6) N=kc

where k represents the grain boundary absorption coefficient. This simplified boundary condition provides a first-order approximation of solute capture behavior at grain boundaries without explicitly resolving atomic-scale grain boundary structures.

Following previous studies, PFZ width was determined based on the region with significant solute depletion relative to the matrix concentration rather than a sharp physical interface [39]:

(7) $$W_{\mathrm{PFZ}}=R-x\left(c/c_{0}=0.7\right)$$

where $x\;\left(c/c_{0}=0.7\right)$ represents the radial position where the solute concentration decreases to 70% of the initial concentration. This criterion provides a unified quantitative parameter for comparing PFZ evolution under different processing conditions.

The initial condition was defined by assuming a homogeneous solute distribution after solution treatment:

(8) $$c\;(r,0)=c_{0}$$

Based on the governing equations of the proposed diffusion–sink model, a qualitative assessment of parameter sensitivity can be made for the key model parameters listed in Table 4. The stage-dependent effective diffusion coefficients ($D_{\mathrm{pre}}^{\mathrm{eff}}$, $D_{\mathrm{reg}}^{\mathrm{eff}}$, and $D_{\mathrm{re}}^{\mathrm{eff}}$) mainly determine the diffusion kinetics and the evolution rate of the Cu concentration profile, whereas the grain boundary absorption coefficient (k) primarily affects the extent of solute depletion near grain boundaries. The rolling strain (ε) and the dislocation enhancement coefficient (α) regulate the effective diffusion coefficient by modifying deformation-enhanced atomic mobility. As indicated by Equations (5)–(7), these parameters mainly affect the diffusion rate and the absolute PFZ width through the effective diffusion coefficient or grain boundary absorption term, without changing the governing diffusion mechanism. Therefore, within the parameter ranges adopted in the present model, the qualitative evolution trend of Cu redistribution and PFZ widening with increasing retrogression time is expected to be preserved, although the absolute PFZ width may vary. Overall, the proposed model is considered suitable for capturing the qualitative evolution tendency of Cu redistribution during RRA treatment.

**Figure 7:**
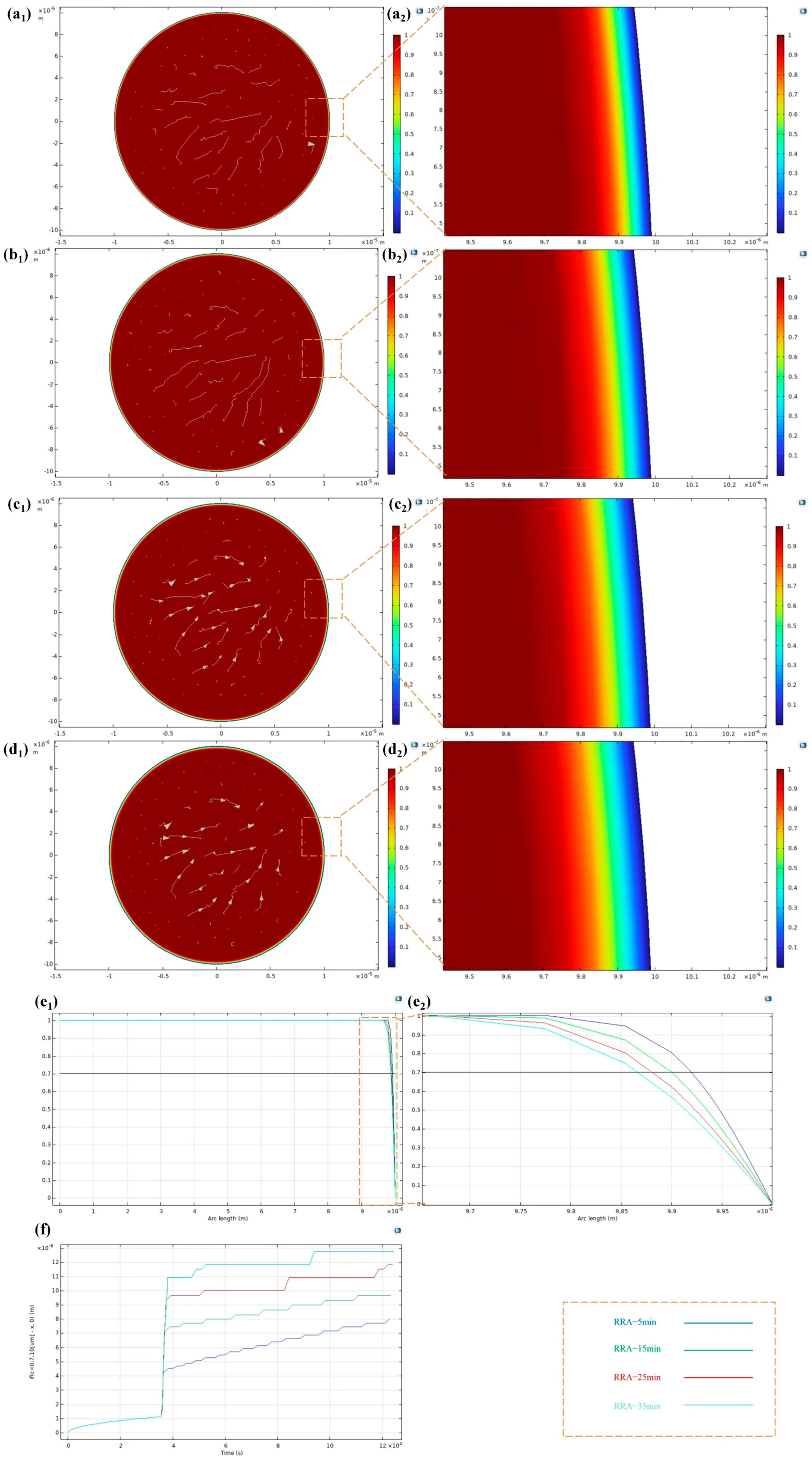
Simulation contour plot of Cu ion concentration in 2195 Al–Cu–Li alloy at the final heat treatment under different retrogression conditions: (**a_1_**,**a_2_**) 5 min, (**b_1_**,**b_2_**) 15 min, (**c_1_**,**c_2_**) 25 min, (**d_1_**,**d_2_**) 35 min, Cu ion concentration profile in Al–Cu–Li alloy at the final heat treatment stage (**e_1_**,**e_2_**), Simulation of PFZ width evolution in 2195 Al–Cu–Li alloy during RRA processing (**f**).

Figure 7 presents the simulated Cu diffusion behavior and PFZ evolution in 2195 Al–Cu–Li alloy during the retrogression stage of RRA treatment. The Cu concentration distributions at different retrogression times are shown in Figure 7a_1_–d_2_. With increasing retrogression time, Cu atoms continuously diffuse from regions near the grain boundary into the surrounding matrix and are absorbed by the grain boundary region, indicating the gradual expansion of Cu-depleted regions on both sides of the grain boundary. Consequently, the PFZ width exhibits a continuous increasing trend. The concentration profiles in Figure 7e_1_,e_2_ further show that the Cu concentration gradient gradually decreases with prolonged retrogression time, reflecting enhanced solute redistribution and reduced compositional differences between the grain boundary region and matrix.

Figure 7f further illustrates the dynamic evolution of PFZ width during RRA treatment. The simulation suggests that PFZ evolution mainly occurs during the retrogression stage. The calculated PFZ widths were 80.0 nm, 96.6 nm, 118.4 nm, and 127.6 nm for RRA-5 min, RRA-15 min, RRA-25 min, and RRA-35 min conditions, respectively. From the proposed Cu diffusion–grain boundary absorption model, shorter retrogression times are associated with limited Cu redistribution and reduced PFZ widening. This microstructural state may help maintain grain boundary integrity, reduce local electrochemical heterogeneity, and mitigate micro-galvanic corrosion and IGC susceptibility. Combined with the experimental results, these findings suggest a favorable strength–corrosion resistance balance for the RRA-5 min and RRA-15 min conditions.

With prolonged retrogression, the model predicts enhanced Cu redistribution and increased solute depletion near grain boundaries, which may contribute to the formation of corrosion-sensitive regions. Meanwhile, moderate PFZ development could potentially alleviate local strain concentration and improve deformation compatibility. Thus, PFZ evolution during RRA treatment represents a balance between corrosion resistance and mechanical performance. The simulated PFZ evolution trend agrees with reported correlations between PFZ characteristics, mechanical properties, and corrosion behavior [10,40], providing qualitative insights into Cu redistribution and PFZ evolution under the investigated RRA processing conditions.

### 3.4. Corrosion Mechanism

**Figure 8:**
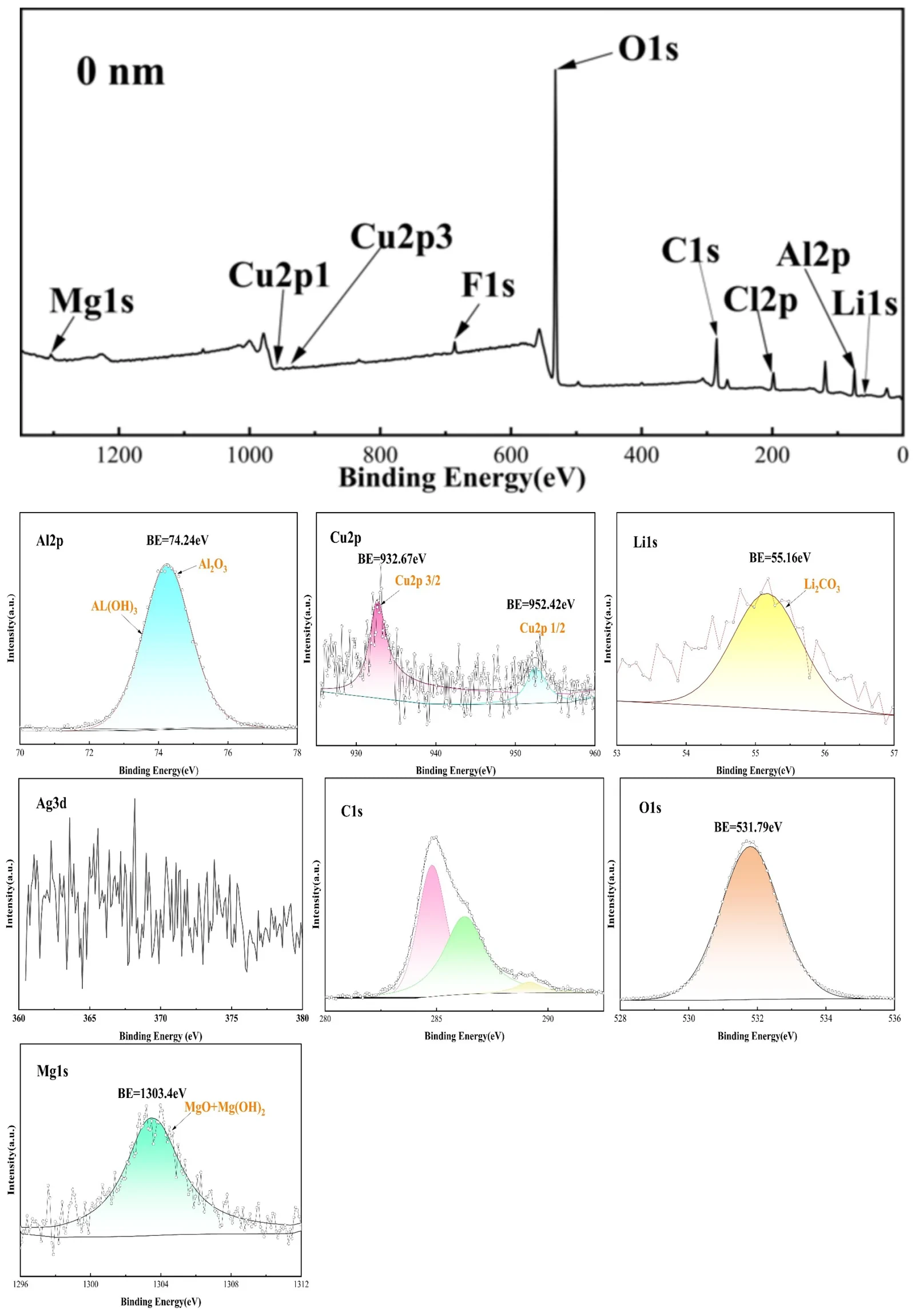
XPS analysis of 2195 Al–Cu–Li alloy at 0 nm depth.

**Figure 9:**
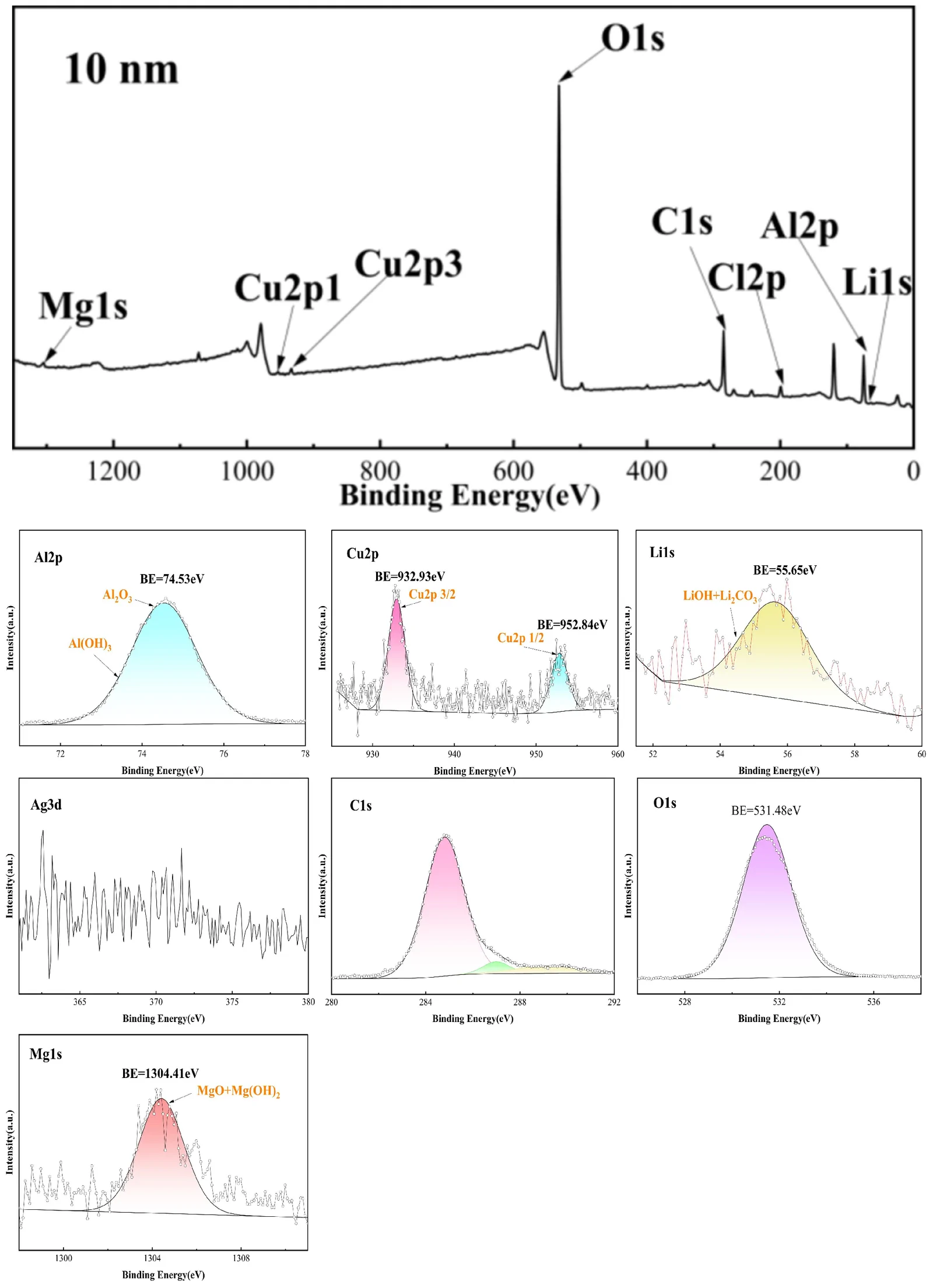
XPS analysis of 2195 Al–Cu–Li alloy at 10 nm depth.

**Figure 10:**
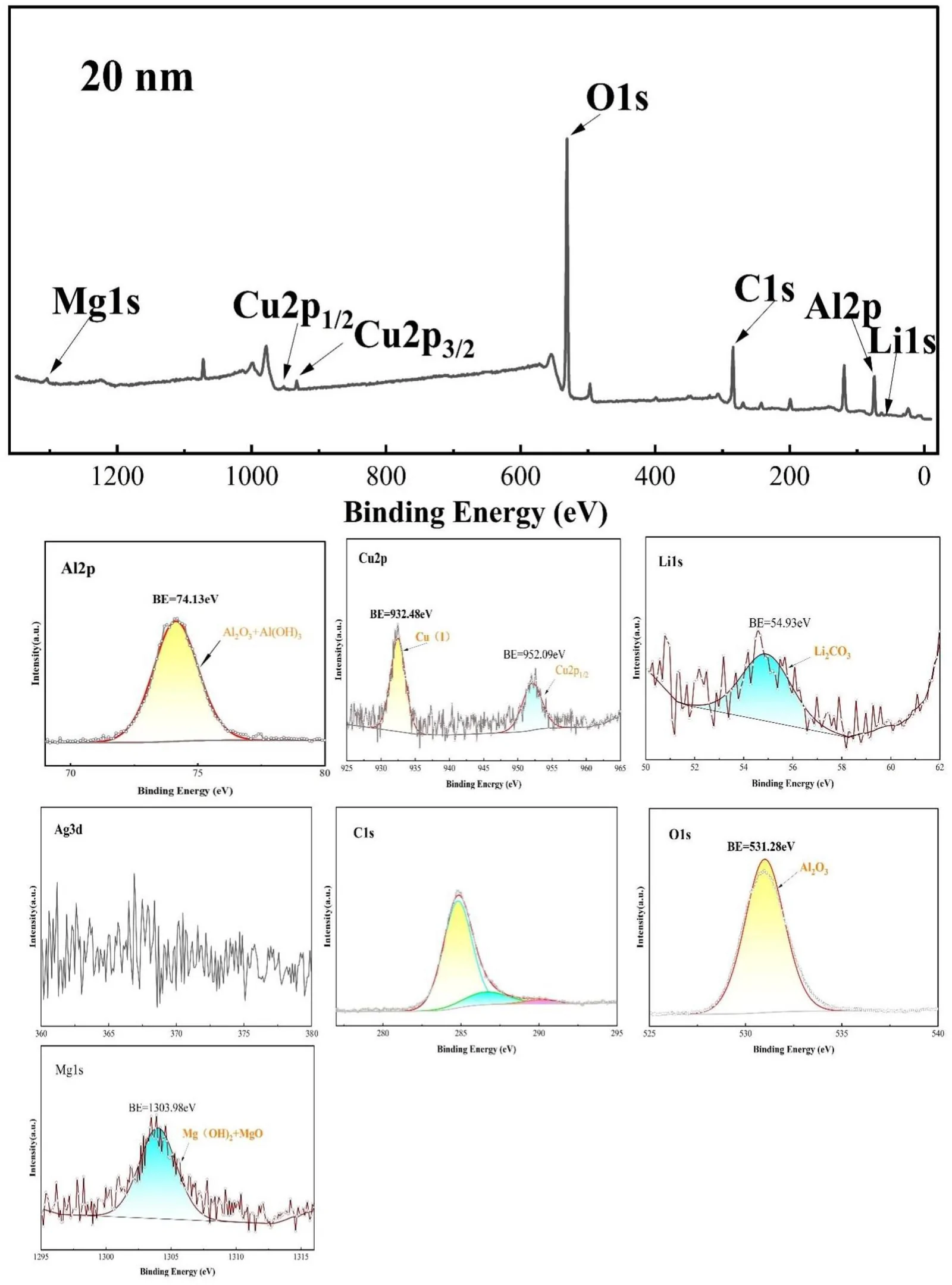
XPS analysis of 2195 Al–Cu–Li alloy at 20 nm depth.

**Figure 11:**
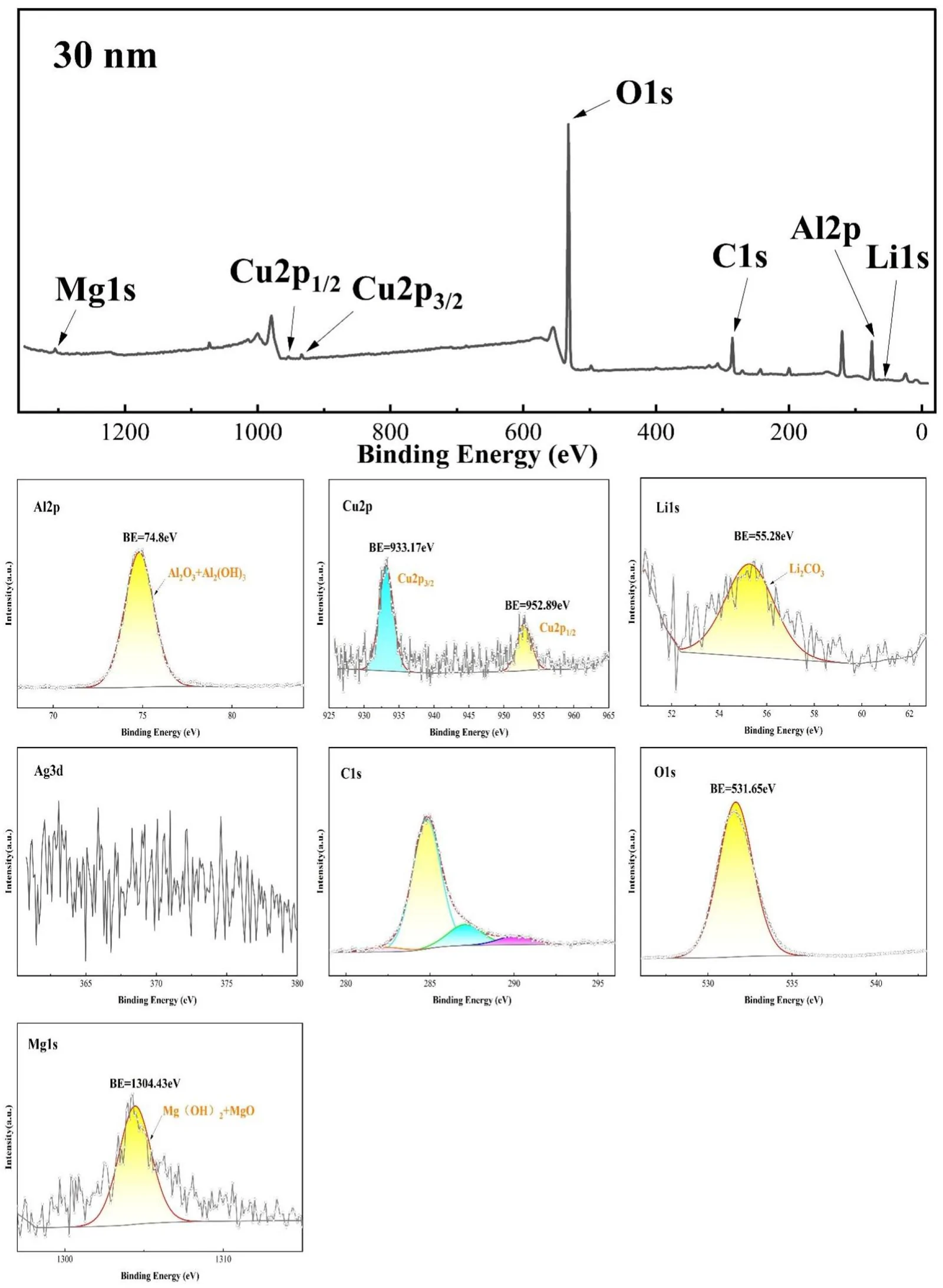
XPS analysis of 2195 Al–Cu–Li alloy at 30 nm depth.

Figure 8, Figure 9, Figure 10 and Figure 11 show the XPS spectra of the 2195 Al–Cu–Li alloy after electrochemical corrosion in 3.5 wt.% NaCl solution at different sputtering depths ranging from 0 to 30 nm. The survey spectra indicate that the corroded surface mainly consists of characteristic peaks corresponding to Al 2p, Li 1s, Mg 1s, Cu 2p, Ag 3d, O 1s, and C 1s. The high-resolution spectra were further deconvoluted to identify the chemical states of the corrosion products. The Al 2p peak located at approximately 74 eV corresponds to Al-containing oxides and hydroxides. Previous studies have reported that Al_2_O_3_ and Al(OH)_3_ exhibit similar binding energies in this region, suggesting that the corrosion products consist of a mixture of these two compounds [41]. The characteristic peak near 55 eV in the Li 1s spectrum suggests the formation of Li-containing corrosion products, with Li_2_CO_3_ identified as the predominant corrosion product [42]. The Mg 1s peak is mainly attributed to Mg(OH)_2_ accompanied by a small amount of MgO [43]. The Cu 2p peaks located at approximately 933 eV (Cu 2p_3/2_) and 952 eV (Cu 2p_1/2_) indicate the presence of metallic Cu species within the corrosion film [44,45]. No obvious fitting peak was observed in the Ag 3d spectra, suggesting that the Ag concentration was below the detection limit or remained extremely low in the analyzed surface region. The O 1s and C 1s signals mainly originate from oxide/hydroxide species and carbonate species, together with surface-adsorbed carbon contamination.

Based on the above analysis, the corrosion product film formed on the 2195 Al–Cu–Li alloy surface mainly consists of Al–Li–Mg–Cu–Ag-related compounds, including Al_2_O_3_, Al(OH)_3_, Li_2_CO_3_, Mg(OH)_2_, and a small amount of MgO, accompanied by localized Cu enrichment and trace Ag accumulation. Generally, pitting corrosion preferentially initiates at second-phase particles or chemically heterogeneous regions, where localized micro-galvanic coupling promotes electrochemical reactions and facilitates the redistribution and enrichment of relatively noble elements such as Cu.

**Figure 12:**
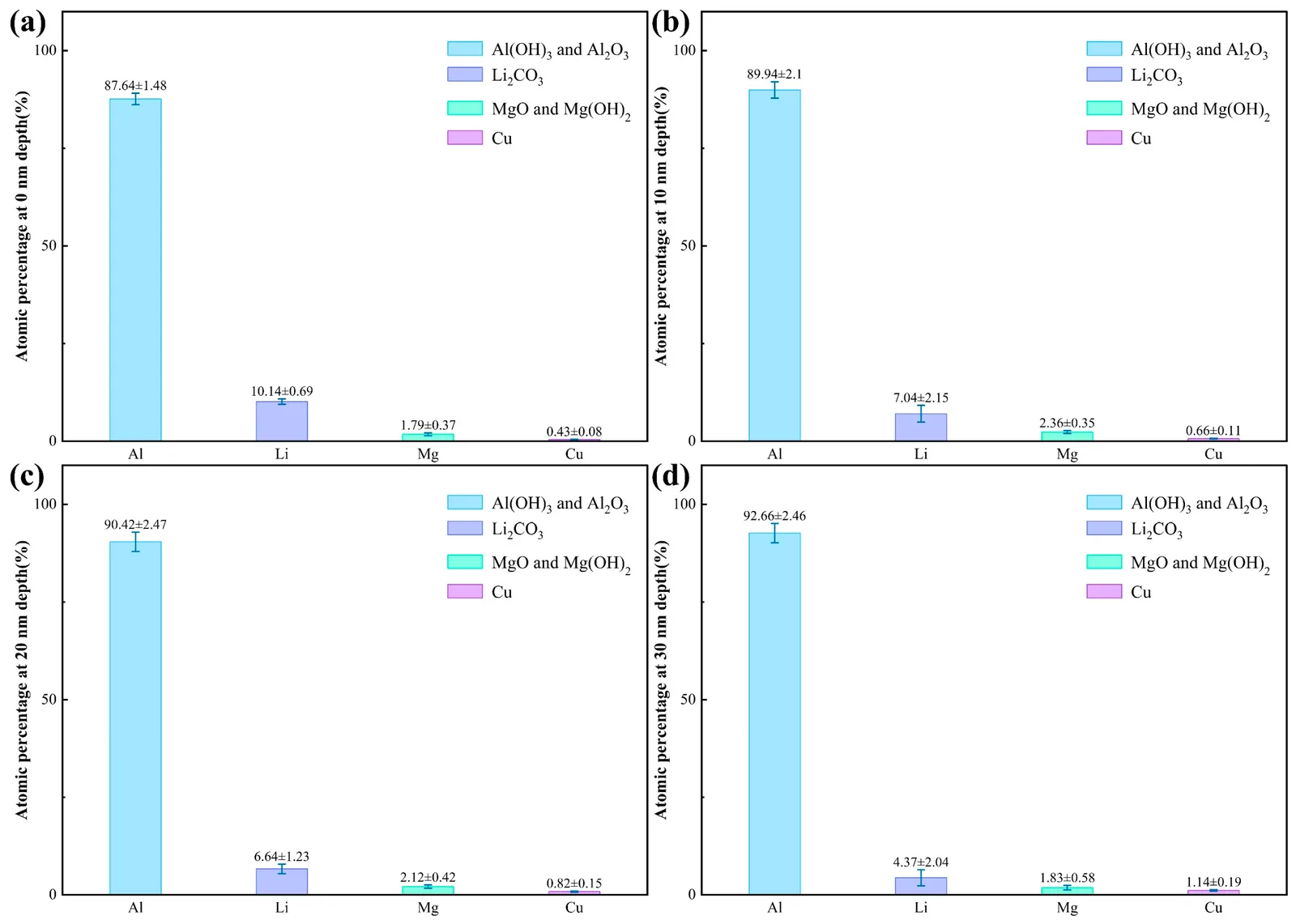
Atomic percentage in the 2195 Al–Cu–Li alloy at 0 nm depth (**a**), 10 nm depth (**b**). 20 nm depth (**c**), 30 nm depth (**d**).

According to the elemental distribution results at different sputtering depths (0–30 nm) shown in Figure 12, the corrosion product film exhibits significant compositional evolution with increasing sputtering depth. The fraction of Al_2_O_3_ gradually increases and becomes the dominant phase, whereas Li_2_CO_3_ continuously decreases, suggesting that Li-containing carbonate/hydroxide species are mainly concentrated in the outer region of the corrosion film. The contents of Mg(OH)_2_ and MgO remain relatively low with slight fluctuations, suggesting their heterogeneous distribution within the surface layer.

**Figure 13:**
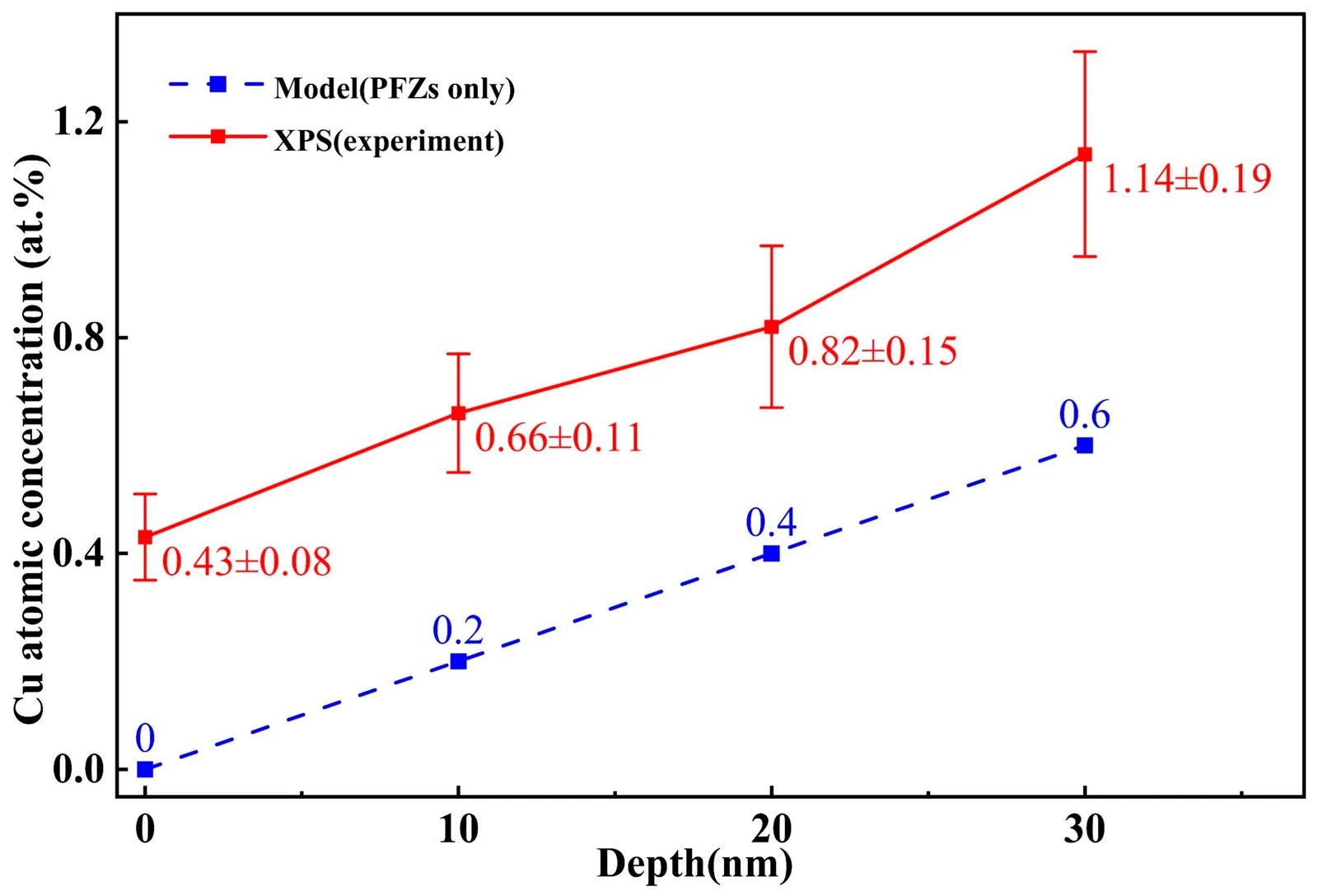
Comparison of simulated and experimental Cu concentration profiles revealing Cu redistribution during RRA treatment.

According to Table 1, the bulk Cu content of the 2195 Al–Cu–Li alloy is approximately 3.9 wt.%. However, quantitative XPS analysis reveals that the relative Cu concentration within the corrosion film is significantly lower than that in the bulk alloy and gradually increases with increasing sputtering depth. This behavior indicates that Cu is not uniformly incorporated into the surface corrosion products but undergoes pronounced interfacial redistribution and selective depletion during corrosion. Considering the microstructural characteristics of the alloy, the observed Cu depletion near the surface can be associated with preferential corrosion behavior in precipitate-depleted regions, such as PFZs, formed during the pre-treatment and RRA process. In PFZ regions, the reduced density of strengthening precipitates and solute depletion near grain boundaries can increase local electrochemical activity, promoting selective Cu dissolution during the initial corrosion stage or migration toward subsurface regions. Consequently, the Cu signal detected in the outer corrosion film is significantly reduced. These observations imply that PFZ regions not only contribute to localized corrosion but also regulate the spatial redistribution of alloying elements, thereby affecting the compositional evolution and interfacial stability of the corrosion product film. The Ag signal remains weak throughout the sputtering process, indicating only limited Ag enrichment mainly within the outermost surface region. Throughout the investigated sputtering depth range, the Cu concentration measured by XPS exhibits a consistent increasing trend with the simulation results, with both showing gradual Cu enrichment as the sputtering depth increases. This agreement indicates that the proposed diffusion–sink model reasonably captures the overall tendency of Cu redistribution during RRA treatment. Consistent with the simulated PFZ evolution, the XPS depth profiles suggest progressive Cu redistribution from the outer corrosion layer toward the subsurface region, supporting the qualitative prediction of solute redistribution during retrogression. As illustrated in Figure 13, however, the experimentally measured Cu concentrations are higher than the simulated values. This discrepancy mainly arises from the simplified assumptions of the present model, which considers only the redistribution of dissolved Cu atoms within the supersaturated Al matrix. In contrast, the actual alloy surface contains Cu-containing T_1_ precipitates (Al_2_CuLi), interfacial Cu segregation, and chemically heterogeneous corrosion products, all of which contribute additional Cu signals during XPS analysis [46]. Therefore, although the model cannot quantitatively reproduce the absolute Cu concentration measured experimentally, the consistent evolution trend between simulation and XPS supports its applicability for describing the qualitative redistribution behavior of Cu during RRA treatment. Furthermore, Cu redistribution and the associated PFZ evolution are considered contributing factors to the development of electrochemical heterogeneity; however, the observed corrosion behavior is also influenced by corrosion product film evolution, precipitate characteristics, and localized micro-galvanic effects. Accordingly, the proposed mechanism should be regarded as a qualitative interpretation supported by both simulation and experimental observations rather than a definitive description of the corrosion process.

The simulation suggests that Cu redistribution remains predominantly diffusion-controlled within the investigated retrogression range (5–35 min). Accordingly, prolonged retrogression is likely to promote Cu migration toward grain boundaries, contributing to PFZ widening. It should be noted that the present diffusion–sink model is mainly developed to capture the evolution tendency of Cu redistribution and PFZ formation during RRA treatment rather than to quantitatively predict the exact PFZ dimensions. Due to the lack of direct microstructural characterization, further studies combining TEM and other advanced characterization techniques will be conducted to validate the PFZ evolution and precipitates.

**Figure 14:**
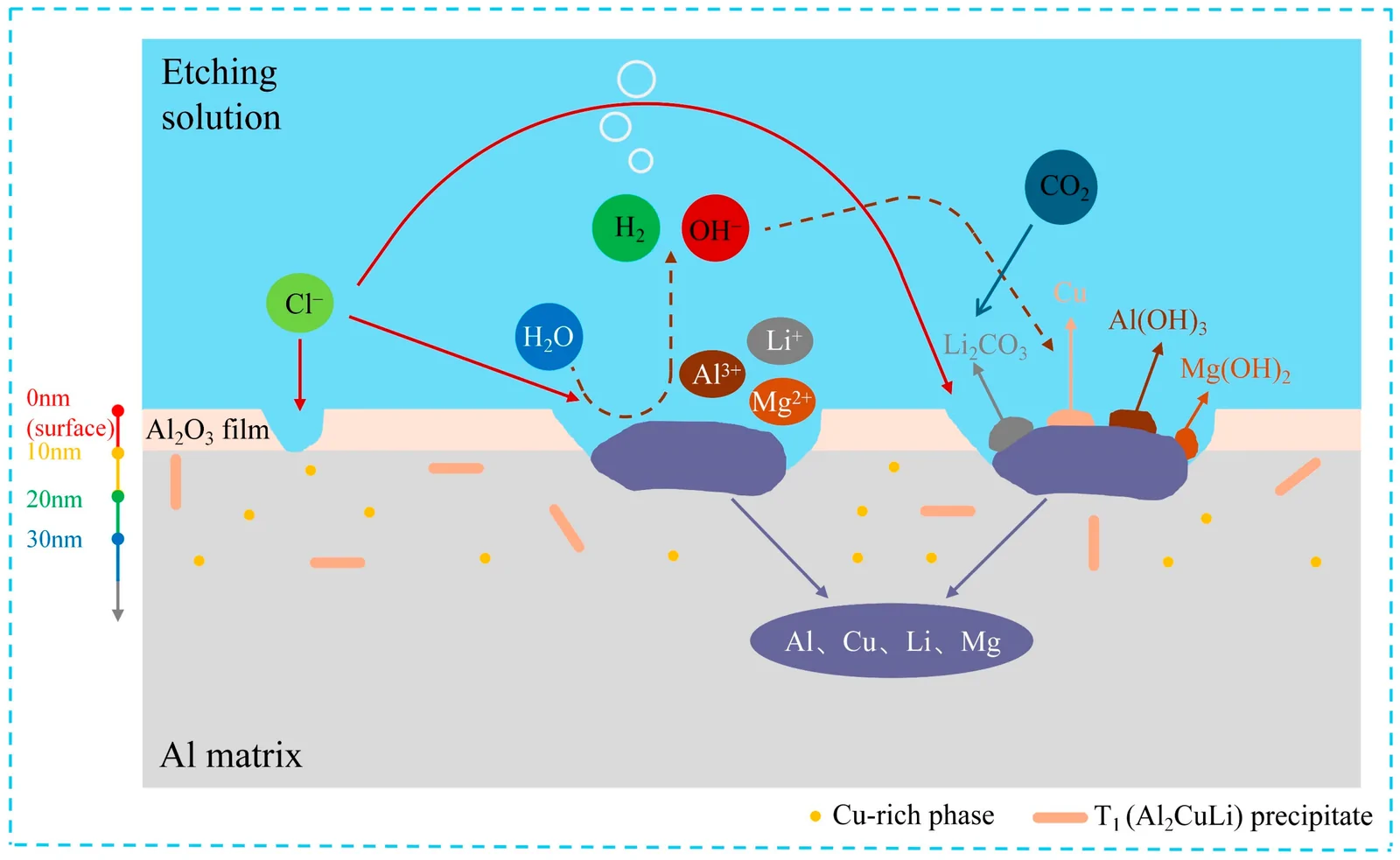
Corrosion mechanism of 2195 Al–Cu–Li alloy in NaCl solution.

As illustrated in Figure 14, during electrochemical corrosion in 3.5 wt.% NaCl solution, water molecules and Cl^−^ ions are initially adsorbed onto the surface of the 2195 Al–Cu–Li alloy, forming a thin electrolyte layer. Due to its strong penetration capability, Cl^−^ ions preferentially accumulate at defect sites within the Al_2_O_3_ passive film and promote localized dissolution of the oxide layer, resulting in the breakdown of the originally continuous and protective passive film.

Meanwhile, the microstructural constituents generated after RRA treatment, including T_1_ (Al_2_CuLi) phases, θ′ (Al_2_Cu) phases, and minor Cu/Ag-rich regions, can form localized micro-galvanic couples with the Al matrix. These heterogeneous electrochemical sites accelerate the anodic dissolution of the surrounding aluminum matrix, leading to the preferential initiation of localized corrosion at second-phase particles, grain boundary regions, and PFZs.

The above corrosion process represents a typical electrochemical corrosion mechanism. In the NaCl electrolyte environment, a complex corrosion product film composed of Al–Li–Mg–Cu–Ag-related compounds is gradually formed on the alloy surface, including Al_2_O_3_, Li_2_CO_3_, MgO, Cu, Mg(OH)_2_, and Al(OH)_3_. Under the applied electrochemical potential, the surface passive film undergoes a continuous dynamic equilibrium between formation and rupture. This repeated breakdown and regeneration process induces localized electrochemical polarization and redistribution of interfacial charges, thereby sustaining the corrosion reactions, as described by Equations (9)–(11):

2Al + 3O^2−^ → Al_2_O_3_ + 6e^−^ (9)

2Li + CO_3_^2−^ → Li_2_CO_3_ + 2e^−^ (10)

Mg + O^2−^ → MgO + 2e^−^ (11)

During the electrochemical corrosion process, the main anodic and cathodic reactions occurring on the surface of the 2195 Al–Cu–Li alloy can be described as follows:

Anodic reaction:

Al → Al^3+^ + 3e^−^ (12)

Li → Li^+^ + e^−^ (13)

Mg → Mg^2+^ + 2e^−^ (14)

Cathodic reaction:

O_2_ + 2H_2_O + 4e^−^ → 4OH^−^ (15)

2H_2_O + 2e^−^ → 2OH^−^ + H_2_ (16)

Subsequently, corrosion products are formed under the locally alkaline environment as follows:

Al^3+^ + 3OH^−^ → Al(OH)_3_ (17)

Mg^2+^ + 2OH^−^ → Mg(OH)_2_ (18)

2Li^+^ + 2OH^−^ + CO_2_ → Li_2_CO_3_ + H_2_O (19)

As corrosion proceeds, Al undergoes preferential dissolution, generating Al^3+^ ions. Meanwhile, Li within the T_1_ phase exhibits a relatively lower electrode potential and higher electrochemical activity, resulting in preferential dissolution and the formation of Li^+^ ions. In the cathodic regions, oxygen reduction reactions dominate, accompanied by the participation of water molecules to produce OH^−^ ions, leading to a gradual increase in the local pH within corrosion pits.

Due to its relatively low electrochemical potential and high chemical activity compared with the Al matrix and precipitates, Mg is prone to preferential anodic dissolution in the NaCl environment, entering the electrolyte or corrosion product film in the form of Mg^2+^ ions. As Al^3+^, Li^+^, and Mg^2+^ continuously migrate into the corrosion film, they react with OH^−^ ions and dissolved CO_2_ from the surrounding environment, forming corrosion products including Al(OH)_3_, Mg(OH)_2_, and Li_2_CO_3_. Among these products, Li-containing compounds tend to preferentially accumulate in the outer region of the corrosion film due to their high chemical activity, whereas Al_2_O_3_ gradually enriches in the inner layer and contributes to the formation of a relatively stable protective barrier.

Meanwhile, Cu and trace Ag exhibit higher corrosion potentials and are less susceptible to dissolution during corrosion. Instead, they gradually accumulate in localized regions, forming Cu-rich layers that further modify the local electrochemical behavior and facilitate surface repassivation.

Overall, the corrosion behavior of the 2195 Al–Cu–Li alloy in NaCl solution can be associated with several coupled processes, including passive film degradation, second-phase-induced micro-galvanic effects, selective dissolution of active elements, corrosion product accumulation, and Cu enrichment during surface film evolution. Based on the XPS results, the corrosion products mainly consisted of Al oxides/hydroxides, Li- and Mg-containing compounds, and Cu-enriched species, indicating the dissolution of reactive elements and subsequent surface film reconstruction. These processes contribute to localized electrochemical heterogeneity. However, these relationships should be regarded as qualitative interpretations rather than definitive mechanistic evidence, because the present study does not directly resolve the nanoscale evolution of precipitates and grain-boundary structures. The proposed corrosion scenario is therefore primarily supported by the observed corrosion morphologies, XPS depth profiles, corrosion-product compositions, and the qualitative trends obtained from the diffusion–sink model.

For samples subjected to shorter retrogression times, the limited thermal exposure is likely to preserve a more stable precipitation structure and restrict excessive solute redistribution near grain boundaries. Together with the experimental observations, this microstructural state may help reduce local electrochemical heterogeneity between grain boundaries and the matrix, thereby contributing to the improved corrosion resistance observed in the present study.

## 4. Conclusions

In this study, solution treatment, 5% room-temperature rolling, and RRA treatment were combined to regulate the microstructural evolution of the 2195 Al–Cu–Li alloys. The effects of RRA retrogression time on microstructure, corrosion behavior, and strength–corrosion synergy were systematically investigated. Under the investigated processing conditions, a retrogression time of 5 min provided the most favorable strength–corrosion synergy by balancing mechanical strength and corrosion resistance. The main conclusions are as follows:

(1) With increasing RRA time from 5 to 35 min, the alloy gradually transforms from a high-strength state to a more ductile condition. Short-term retrogression enhances dislocation strengthening, resulting in the highest strength for the RRA-5 min sample. Prolonged retrogression promotes microstructural coarsening and grain boundary softening, reducing strengthening efficiency while improving plastic deformation capability. Thus, RRA time effectively regulates the strength–ductility balance.
(2) The corrosion resistance decreases overall with increasing RRA time. The RRA-5 min sample exhibits the best corrosion resistance, with the highest polarization resistance (19,605 Ω·cm^2^) and the lowest corrosion current density (3.89 × 10^−7^ A·cm^−2^). Short-term retrogression is associated with stable grain boundary characteristics, which may contribute to reduced electrochemical heterogeneity and micro-galvanic corrosion. In contrast, prolonged retrogression leads to enhanced Cu redistribution, which could increase corrosion susceptibility.
(3) A multilayer corrosion product film composed of Al_2_O_3_, Al(OH)_3_, Li/Mg-containing compounds, and Cu-enriched species forms after corrosion. With increasing sputtering depth, Li-containing products decrease, while Al-containing compounds become dominant and Cu gradually recovers, revealing selective dissolution and elemental redistribution. Microstructural heterogeneity promotes localized micro-galvanic corrosion, contributing to corrosion initiation.
(4) Based on a simplified Cu diffusion–grain boundary absorption model combined with experimental observations, Cu redistribution was identified as a key factor governing PFZ evolution during RRA treatment. The simulated results suggest that prolonged retrogression promotes Cu migration toward grain boundaries, which is likely associated with PFZ widening and increased corrosion susceptibility. Although the present model simplifies precipitate evolution and grain boundary characteristics, it provides qualitative insights into the evolution tendency of Cu redistribution and PFZ development during RRA treatment. Further validation by direct nanoscale microstructural characterization is needed in future work.

## Figures and Tables

**Table 1:** Chemical composition of the studied 2195 Al–Cu–Li alloy (wt.%).

Cu	Mg	Li	Zr	Ag	Fe	Si	Ti	Al
3.90	0.50	1.01	0.12	0.36	0.022	0.019	0.059	Bal.

**Table 2:** Corrosion potential and corrosion current of 2195 Al–Cu–Li alloy.

Sample	E_corr_ (V)	I_corr_ (A/cm^2^)
RRA-5 min	−0.7933	3.8878 × 10^−7^
RRA-15 min	−0.8120	4.1381 × 10^−7^
RRA-25 min	−0.86595	1.9009 × 10^−6^
RRA-35 min	−0.6494	1.4919 × 10^−6^

**Table 3:** Impedance parameters for 2195 Al–Cu–Li alloy.

Sample	Rs (Ω·cm^2^)	CPEf-T (F cm^−2^)	CPEf-P	Rf (Ω·cm^2^)	CPEp-T (F cm^−2^)	CPEp-P	Rp (Ω·cm^2^)
RRA-5 min	21.81	1.5992 × 10^−5^	0.89045	5287	1.9475 × 10^−4^	0.83707	19,605
RRA-15 min	6.517	2.6201 × 10^−5^	0.83859	2711	5.3265 × 10^−4^	1.072	4013
RRA-25 min	5.492	7.8645 × 10^−5^	0.85545	8987	3.1781 × 10^−3^	1.112	8896
RRA-35 min	4.932	1.1327 × 10^−4^	0.71091	2399	6.0403 × 10^−3^	1.107	2683

**Table 4:** Material properties used in the simulation model.

Parameters	Values	Role
$D_{\mathrm{pre}}^{\mathrm{eff}}$	5 × 10^−22^ m^2^/s	Effective diffusion coefficient during pre-aging at 165 °C
$D_{\mathrm{reg}}^{\mathrm{eff}}$	8 × 10^−19^ m^2^/s	Effective Cu diffusion coefficient during retrogression at 210 °C
$D_{\mathrm{re}}^{\mathrm{eff}}$	7 × 10^−21^ m^2^/s	Effective Cu diffusion coefficient during re-aging at 175 °C
k	10^−5^ m/s	Grain boundary absorption strength
ε	0.05	Rolling strain
α	50	Dislocation enhancement coefficient

## Data Availability

The original contributions presented in this study are included in the article. Further inquiries can be directed to the corresponding author.
